# Robust Measures of Image-Registration-Derived Lung Biomechanics in SPIROMICS

**DOI:** 10.3390/jimaging8110309

**Published:** 2022-11-16

**Authors:** Yue Pan, Di Wang, Muhammad F. A. Chaudhary, Wei Shao, Sarah E. Gerard, Oguz C. Durumeric, Surya P. Bhatt, R. Graham Barr, Eric A. Hoffman, Joseph M. Reinhardt, Gary E. Christensen

**Affiliations:** 1 Department of Electrical and Computer Engineering, University of Iowa, Iowa City, IA 52242, USA; 2 The Roy J. Carver Department of Biomedical Engineering, University of Iowa, Iowa City, IA 52242, USA; 3 Department of Electrical and Computer Engineering, University of Florida, Gainesville, FL 32611, USA; 4 Department of Mathematics, University of Iowa, Iowa City, IA 52242, USA; 5 UAB Lung Imaging Core, University of Alabama at Birmingham, Birmingham, AL 35294, USA; 6 Departments of Medicine and Epidemiology, Columbia University Medical Center, New York, NY 10032, USA; 7 Department of Radiology, University of Iowa, Iowa City, IA 52242, USA

**Keywords:** image registration, lung, biomechanics, COPD, SPIROMICS

## Abstract

Chronic obstructive pulmonary disease (COPD) is an umbrella term used to define a collection of inflammatory lung diseases that cause airflow obstruction and severe damage to the lung parenchyma. This study investigated the robustness of image-registration-based local biomechanical properties of the lung in individuals with COPD as a function of Global Initiative for Chronic Obstructive Lung Disease (GOLD) stage. Image registration was used to estimate the pointwise correspondences between the inspiration (total lung capacity) and expiration (residual volume) computed tomography (CT) images of the lung for each subject. In total, three biomechanical measures were computed from the correspondence map: the Jacobian determinant; the anisotropic deformation index (ADI); and the slab-rod index (SRI). CT scans from 245 subjects with varying GOLD stages were analyzed from the SubPopulations and InteRmediate Outcome Measures In COPD Study (SPIROMICS). Results show monotonic increasing or decreasing trends in the three biomechanical measures as a function of GOLD stage for the entire lung and on a lobe-by-lobe basis. Furthermore, these trends held across all five image registration algorithms. The consistency of the five image registration algorithms on a per individual basis is shown using Bland–Altman plots.

## 1. Introduction

Chronic obstructive pulmonary disease (COPD) refers to a collection of inflammatory lung diseases including chronic bronchitis and emphysema [1]. COPD continues to be the third leading cause of death worldwide [1], with an ever-increasing disease burden [2]. People with COPD may experience high morbidity, high healthcare costs, poor quality of life, activity limitation, and exacerbations [3]. Although COPD is not curable, available treatments can help relieve symptoms, improve exercise capacity, improve the quality of life, reduce the risk of death, and reduce the cost of healthcare [4]. Spirometry—a common pulmonary function test—is currently the gold standard for diagnosing and staging COPD. Based on spirometry, the Global Initiative for Chronic Obstructive Lung Disease (GOLD) recommends grading the disease into four categories, ranging from GOLD 1 to GOLD 4 [5]. However, spirometry alone cannot capture the heterogeneous manifestations of COPD, which calls for better diagnostic methods. Towards advancing our understanding of the disease, several multi-center studies such as SPIROMICS [6] and the Genetic Epidemiology of COPD, COPDGene [7] are underway. Along with spirometry, these studies rely on computed tomography (CT) scans of the subjects, which have led to the development of several imaging biomarkers for characterizing COPD.

Biomechanical properties of the lung can be used to characterize lung function and are important because they provide information on whether or not the lung is functioning normally or abnormally. One way to indirectly measure the biomechanical properties of the lung at the local level is to analyze the pointwise correspondences between inspiration and expiration CT image volumes. This can be achieved using image registration. Image registration is used to find the point-to-point correspondence between two images in the form of a transformation or deformation vector field (DVF). Biomechanical measurements (also called biomechanical biomarkers) can then be extracted from the transformation to describe the local expansion or contraction of the lung during the breathing cycle.

A complementary approach to extracting biomarkers from CT images is to compute CT intensity-based disease biomarkers. Intensity-based disease biomarkers are computed from CT image intensities with or without using image registration. These methods extract biomarkers at each CT voxel based on the voxel intensity or based on the intensity pattern (texture) of a local region of voxels. Intensity-based biomarkers are widely used to infer disease patterns, but they are susceptible to image noise and do not provide biomechanical measurements. An example of this type of analysis includes defining emphysematous regions by thresholding the inspiratory scan below −950 Hounsfield units (HU), while the cut-off for air-trapping regions in an expiratory scan is −856 HU. This technique is often referred to as CT densitometry, and is prone to noise arising from changes in volume of acquisition, CT dosage, and scanning parameters [8,9,10]. To overcome these challenges, texture-based CT analysis methods have been developed to assess parenchymal integrity and its relation to disease progression. Sørensen et al. [11] developed a CT texture classification method to assess COPD. A similar method was used to assess pulmonary emphysema using local binary patterns [12]. These methods rely on globally annotated CTs, and lack local functional information related to parenchymal disintegration. A texture-based approach called CALIPER was used to quantify interstitial lung abnormalities and showed good correlations between the fraction of abnormal lung texture and lung functional measurements [13]. More recently, a deep convolutional neural network (CNN) was employed for staging COPD and predicting disease progression [14]. Although this approach was able to learn effective representations associated with COPD severity and progression, it failed to provide insights into the regional distribution of lung function abnormalities.

Several image-registration-based biomechanical biomarkers have been derived from the DVF to understand, diagnose, and stage COPD. Galbàn et al. [15] used CT intensity and image registration to define the parametric response mapping (PRM), a two-dimensional histogram that measures the amounts of normal tissue, small airways disease, and emphysema within the lung. Amelon et al. [16] extracted three biomechanical indices from the DVF of pulmonary inspiration-to-expiration registration to characterize local and global lung deformation: the Jacobian determinant (*J*); the anisotropic deformation index (ADI), and the slab-rod index (SRI) [16]. Bodduluri et al. [17] evaluated the predictive performance of *J*, ADI, and strain extracted from image registration and compared their performance with conventional CT texture and densitometry [18,19] features. They demonstrated that for a complex task such as COPD severity prediction, the biomechanical features performed better than the conventional CT texture and density features. The current study differs from that of Bodduluri et al. in many ways. The work of Bodduluri et al. analyzed data from the Genetic Epidemiology of COPD (COPDGene) study whereas the current study analyzes data from the SPIROMICS study. The SPIROMICS study collected CT scans at total lung capacity (TLC) and residual volume (RV) whereas the COPDGene study collected CT scans at functional residual capacity (FRC), as opposed to RV. Registering TLC to RV CT images presents a more challenging problem than registering TLC to FRC because there is a comparatively larger shape change from TLC to RV than TLC to FRC. The current study extends the work of Bodduluri et al. from a single image-registration method to multiple image-registration algorithms. Finally, the current study analyzes nearly twice as many data sets compared to that of the previous study by Bodduluri et al.

In another study, Bhatt et al. [20] showed an agreement between registration-based mechanics and spirometric measures of lung function and concluded that dual volume (inspiration–expiration) biomechanical measures are better indicators of declining lung function and emphysema than spirometry alone. Another important work by Bodduluri et al. [21] identified the mean of *J* to be significantly associated with different measures of lung function including forced expiratory volume in 1 s (FEV${}_{1}$), emphysema, and six-minute walk distance (6MWD). In a recent work by Pan et al. [22], correlations between air-trapping regions or emphysema regions with mean *J* and mean ADI were found. This work showed that the trends of mean *J* and ADI decreased monotonically as GOLD stages increased for one registration algorithm. While the aforementioned studies point towards clinical effectiveness of registration-based mechanics, they were limited to global features derived from these biomechanical measures. Recently, Chaudhary et al. [23] showed the predictive capabilities of global statistical features extracted from *J*, ADI, and SRI across the four registration methods used in this study. The work by Chaudhary et al. [23] differs from the current paper in that it was limited to the development of classification methods for predicting COPD GOLD stage.

Several image registration algorithms have recently been proposed for analyzing CT lung images of COPD subjects, such as the total variation regularization method [24], key-points-based method [25], and Markov random field (MRF)-based discrete optimization [26]. In addition, deep learning image registration techniques bear the potential to perform image registration orders of magnitude faster than traditional iterative image registration techniques [27,28,29,30,31,32].

The goal of the current study was to understand and evaluate the robustness of different lung biomechanical measures across different image registration methods over a varying disease severity. In this study, we compared and contrasted five diverse image registration algorithms with different similarity costs and transformation models to investigate the impact of different algorithms on the biomechanical measures extracted from the DVF. Image registration performance was evaluated using the lung lobe Dice coefficient (LDC) to measure the overlap of the five lobes after registration; the worst 10% surface error (W10SE) to measure how well the lung surfaces aligned after registration; and the vessel tree position error (VTPE) and symmetric closest skeleton error (SCSE) to measure how well the lung vessel trees aligned. The transformations were used to compute the *J*, ADI and SRI measures [16] in order to analyze and characterize the lung function of individuals with COPD at varying GOLD stages.

## 2. Materials and Methods

### 2.1. Data

The CT images used in this study were part of the SubPopulations and Intermediate Outcome Measures in COPD Study (SPIROMICS) with institutional review board (IRB) approval number 201003733 [6]. SPIROMICS is an ongoing prospective cohort study designed to identify novel clinical stratifications in subjects with COPD. CT images were collected at baseline, one-year, three-year, and five-year follow-ups from fourteen university-based clinical centers across the United States [6].

In this study, we analyzed CT images from current and former smokers across varying degrees of disease severity, as defined by GOLD stage. The GOLD disease staging system for COPD ranges from GOLD 1 (mild), to GOLD 2 (moderate), GOLD 3 (severe) and GOLD 4 (very severe) [5]. GOLD 0 subjects are asymptomatic smokers without airflow obstruction but at risk for COPD due to their smoking history [6]. We analyzed the baseline scans of 245 subjects chosen randomly from the 14 clinical sites, with 49 subjects from GOLD 0, 50 subjects from GOLD 1, 49 subjects from GOLD 2, 50 from GOLD 3, 47 subjects from GOLD 4. At each visit, a pair of 3D breath-hold CT scans were acquired; one at total lung capacity (TLC), and the other at residual volume (RV) [33]. The SPIROMICS imaging protocol uses a CT dose index to standardize exposure across scanners in different sites. The slice collimation was set at 0.6 mm, rotation time 0.5 s and pitch to 1.0. Philips B, GE Standard, and Siemens B35 reconstruction kernels were used [33]. The resolution of the CT scans was approximately 0.6×0.6×0.5 mm${}^{3}$, and the image size was 512×512 per slice, with 500 to 600 slices per image. Full details on the scanning protocol are described by Sieren et al. [33].

### 2.2. Preprocessing

**Figure 1:**
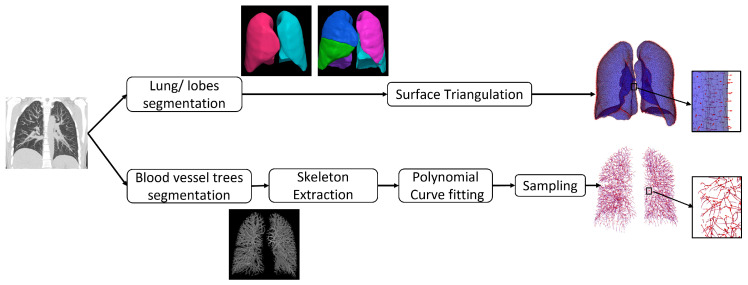
Preprocessing pipeline.

Figure 1 shows the preprocessing pipeline applied to each CT image volume.

All CT image volumes were resampled to be isotropic with spacing 1×1×1 mm${}^{3}$. The image volumes were cropped based on the 3D bounding box containing the union of the lung regions of the inspiration and expiration scans to reduce computer memory requirements and computation time. A multi-resolution convolutional neural network [34] was used to generate a segmentation of the entire lung from the CT volume. The lobes were segmented from the CT volume using the FissureNet deep learning method [35,36]. We used the recommended parameters from the cited papers for the lung and lobe segmentations.

The lobe segmentations were tessellated into triangles using the surface mesh algorithm [37] implemented in the Computational Geometry Algorithms Library (CGAL) (https://www.cgal.org (accessed on 15 September 2017)) that implements the Variational Shape Approximation (VSA) method [38]. The mesh size was set to 2 mm${}^{2}$ with smoothing to give accurate representation of the surfaces. The blood vessel trees were segmented from the CT images using the vesselness filter developed by Jerman et al. [39]. In this method, the eigenvalues of the Hessian matrix are computed from the intensity values of the CT image at each voxel location. Tubular structures are identified at voxel locations that have one near-zero eigenvalue and two non-zero eigenvalues with similar magnitudes. Vessels were detected at different scales by computing the Hessian matrix at different scales. For the vessel segmentation, we started from the parameters recommended in the cited papers, then slightly fine-tuned them. A binary vessel segmentation was computed from the vesselness probability map by thresholding. The threshold was computed using Otsu’s method. Next, the skeletons were extracted from the binary vessel segmentation using the binary 3D thinning algorithm implemented with Insight Toolkit (ITK) in C++ [40] with default parameters. The TLC and RV CT data sets used in this study were collected in register with each other. Therefore, no affine registration was performed in this study before applying the nonrigid image registration algorithms used in this study.

### 2.3. Image Registration Algorithms

We selected five image-registration algorithms to cover a wide range of image registration algorithms. In general, image registration algorithms consist of three major components: an overall cost function to be minimized, a transformation model, and an optimization method. In this study, we focus on the two former components. In general, the overall cost function is a linear combination of a similarity cost and a regularization cost of the transformation. There are various choices for these two components, and therefore different combinations. In terms of similarity cost functions, we can divide them into two major categories: intensity-based and feature-based. Commonly employed intensity-based similarity cost functions used for matching CT scans include, but are not limited to the sum of squared difference (SSD) [41,42], cross correlation (CC) [43], mutual information (MI) [44], and the sum of squared tissue volume difference (SSTVD) [45,46,47]. Examples of intensity-based featured-matching cost functions are the SSD vesselness measure [45], and SSD of lobar segmentations [48] of the lung. For shape-based feature matching, the shapes could be corresponding or non-corresponding points, curves, and surfaces. A popular example is the Iterative Closest Point (ICP) algorithm, which can be used to match point clouds [49], curves (contours), and surfaces [50]. Another group of shape-matching methods adopt the concept of currents and varifolds, initially proposed by Charon et al. [51], and extensively studied by Durrelman et al. [52,53,54,55,56,57]. The idea of applying currents to pulmonary registration was first proposed by Gorbunova et al. [58], and later Pan et al. extended the study by using varifolds representation [59,60]. The advantages of representing shapes for registration purpose with currents and varifolds are (1) point correspondence is not required to define the distance between landmarks, curves, surfaces, and intensity with currents and varifolds representations; and (2) landmarks, curves, surfaces, and intensity can be unified in one framework.

The transformation model plays a critical role in an image registration algorithm since it determines the properties of the DVF. The aim of this study was to register TLC inspiration scans to RV expiration scans of the lungs. Non-rigid image registrations—also often referred to as deformable image registration (DIR)—can be classified based on physical models, interpolation theory models, knowledge-based models and task-specific models [61]. In this manuscript, we classify the transformations as either small or large deformation models. The main differences between small and large deformation models are that the large deformation model allows for more curved particle paths from the moving image to target image, and guarantees a one-to-one correspondence between the moving and target images. The large deformation diffeomorphic metric mapping (LDDMM) model [41] refers to the large deformation model throughout this manuscript. The DVFs that are parameterized by basis functions are categorized into small deformation model, such as Fourier series [42], Thin-Plate spline [62], B-spline [46,63,64,65], etc.

To cover a wide category of image registration algorithms, we selected five algorithms as follows: the Sum-of-Squared-Tissue Volume-Difference (SSTVD) [45], Geodesic Density Regression (GDR) [66,67], Greedy Symmetric Normalization (GSyN) [43], Pulmonary blood Vessel and lobe Surface Varifold (PVSV) [51,57,60], and the Population Learning followed by One Shot Learning (PLOSL) [32].

#### 2.3.1. SSTVD

The SSTVD algorithm was selected as one of the algorithms used because it was developed particularly for registering pulmonary CT and won the Computed Tomography Ventilation Imaging Evaluation 2019 (CTVIE19) Grand Challenge at the American Association of Physicists in Medicine (AAPM) 2019 annual meeting. The SSTVD algorithm is currently being used in the Functional Avoidance Radiation Therapy Clinical trial NCT02843568 and has been used to treat more than 50 subjects in that trial. The SSTVD cost function models the local intensity changes seen in the CT images of the lung due to breathing and provides good correspondence information between inspiration and expiration CT scans.

The overall cost of the SSTVD algorithm is the sum of three terms: (1) the sum of squared tissue volume difference (SSTVD) similarity cost for matching pulmonary CT intensity [46,47], (2) the sum of squared vesselness measure difference (SSVMD) similarity cost for matching lung vessels, and (3) a regularization cost on the cubic B-Splines parameterized DVF to guarantee a smooth and plausible transformation [45]. The main idea behind SSTVD is that lung tissue volume remains relatively constant over the breathing cycle while the volume of air in the lung does not. This method can be considered as a lung-specific intensity-based and feature-based combined small deformation algorithm. The cost function of SSTVD algorithm [22,45,46,47,68,69] is given by:

(1) $$\begin{array}{c} C=\gamma_{1}\frac{1}{\left|\Omega \right|}\int_{\Omega }{\left(I_{f}\left(x\right)-\left|J_{\varphi^{-1}}\left(x\right)\right|I_{m}\left(\varphi^{-1}\left(x\right)\right)\right)}^{2}dx\\ \;\;\;\;\;\;\;+\gamma_{2}\frac{1}{\left|\Omega \right|}\int_{\Omega }{\left(I_{fvm}\left(x\right)-I_{mvm}\left(\varphi^{-1}\left(x\right)\right)\right)}^{2}dx+\gamma_{3}\int_{\Omega }{\left||L\left(u\left(x\right)\right)|\right|}^{2}dx, \end{array}$$

where $I_{f}$ and $I_{m}$ are the fixed and moving tissue density images, respectively; $I_{fvm}$ and $I_{mvm}$ are the fixed and moving vesselness images, respectively; *L* is the linear elasticity differential operator of the type $L={\left(-\alpha \Delta +\gamma \right)}^{\beta }I_{n\times n}$; $\Omega \subset R^{3}$ is the domain of $I_{f}$ and $I_{m}$; The $|J_{\varphi^{-1}}\left(x\right)|$ term is the Jacobian determinant of $\varphi^{-1}$ and used to accommodate intensity changes in the CT images due to changing air content by ensuring the total tissue volume of the lung remains constant; $\varphi^{-1}\left(x\right)$ is the transformation from the fixed to the moving coordinate system; $u\left(x\right)=x-\varphi^{-1}\left(x\right)$ is the displacement field; and $\gamma_{1}$, $\gamma_{2}$ and $\gamma_{3}$ are weights that control the relative importance of each term in the cost function.

#### 2.3.2. GDR

The GDR algorithm was chosen since it is an example of a large deformation image registration algorithm that uses the SSTVD cost function. The SSTVD and GDR image registration algorithms were chosen to compare and contrast the effect of using small vs. large deformation transformation models. The SSTVD and GDR image registrations also differed in that the SSTVD algorithm included the SSVMD similarity method while the GDR did not.

The Geodesic Density Regression (GDR) algorithm [66,67] finds the geodesic path in image space to align pulmonary CT images. The overall cost of the GDR includes the SSTVD term and the regularization term on the velocity fields, which are used to parameterize the DVF as a flow of diffeomorphism. The differences between GDR in this study and the previous SSTVD method are that the GDR algorithm is based on the LDDMM large deformation model [41], and uses the SSTVD similarity cost but does not use the SSVMD similarity cost. GDR can be categorized as a lung-specific intensity-based large deformation model. The cost function of the GDR is given by

(2) $$C=\gamma_{1}\frac{1}{\left|\Omega \right|}\int_{\Omega }{\left(I_{f}\left(x\right)-\left|J_{\varphi^{-1}}\left(x\right)\right|I_{m}\left(\varphi^{-1}\left(x\right)\right)\right)}^{2}dx+\gamma_{2}\int_{0}^{1}{\left||Lv\left(t\right)|\right|}^{2}dt,$$

where the first term is the SSTVD cost function using the same definitions as in Equation (1). The time-varying velocity field v(t) parameterizes the transformation φ by the ordinary differential equation (O.D.E.):

(3) $$\frac{\partial }{\partial t}\phi^{v}\left(x,t\right)=v_{t}\left(\phi_{t}^{v}\left(x\right)\right),$$

where t∈[0,1], $\phi_{0}=Identity$ and $\varphi =\phi_{1}^{v}$ (see [41] for details). The second term in Equation (2) is the regularization cost and is used to find the geodesic path in image space between $I_{f}$ and $I_{m}$. The large deformation model estimates a diffeomorphic transformation φ that guarantees the transformation is smooth with a smooth inverse. A diffeomorphic transformation preserves the topological properties of objects in an image, i.e., connected structures remain connected, disjoint structures remain disjoint, and the smoothness of curves and surfaces are preserved. The relative importance of each term compared to each other are controlled by weights $\gamma_{1}$ and $\gamma_{2}$.

#### 2.3.3. GSyN

The Greedy Symmetric Normalization (GSyN) algorithm [43] is part of the open source ANTs (Advanced Normalization Tools) toolbox [70]. GSyN aligns two images with the deformable diffeomorphic transformation (LDDMM) model [43] in a symmetric scheme. The GSyN algorithm differs from the SSTVD and GDR methods in that it estimates the transformation from moving image to fixed image by deforming both images to the mid-point between the two images. The GSyN algorithm fits into the intensity-based large-deformation model image registration algorithm class. The transformation model of GSyN differs from the GDR algorithm in that GDR uses a time-varying velocity field whereas the GSyN uses a stationary velocity field. The difference is that a stationary velocity field is constant for all time *t* in Equation (3) and therefore uses less parameters to represent the transformation compared to the non-stationary velocity field parameterization and has fewer degrees of freedom than a time-varying velocity field.

The GSyN algorithm used the normalized cross correlation (NCC) similarity cost function compared to the SSTVD similarity cost used by the GDR algorithm. The NCC cost function does not accommodate changes in CT intensity due to breathing in contrast to the SSTVD cost function. This means that the NCC cost function may not always provide the proper correspondences between the moving and target images. To understand this, assume two regions of the parenchyma have the same intensity in the moving image. Next, assume that one region expanded and the other region did not as a result of breathing. The intensity of the expanded region will become darker due to an increase in air filling the regions (i.e., reduced tissue density) and the other region will show no intensity change. The NCC cost is then forced to do its best to match one intensity in the moving image with two different intensities in the target image.

The overall GSyN cost function is defined as

(4) $$C=\gamma_{1}\int_{\Omega }NCC\left(I_{m}\left(\varphi_{m}\left(x\right)\right),I_{f}\left(\varphi_{f}\left(x\right)\right)\right)dx+\gamma_{2}\int_{0}^{0.5}(||Lv_{m}{\left(t\right)||}^{2}+||Lv_{f}{\left(t\right)||}^{2})dt,$$

where *x* is a coordinate of the mid-point coordinate system of $I_{m}$ and $I_{f}$. Note that we use the fixed and moving notation to describe the GSyN method to be consistent with the other registration algorithms even though both images can be thought of as moving. The transformations $\varphi_{m}$ and $\varphi_{f}$ are from the mid-point coordinate system to the coordinate systems of $I_{m}$ and $I_{f}$, respectively. Equation (3) is used to parameterize $\varphi_{m}$ and $\varphi_{f}$ by $v_{m}$ and $v_{f}$, respectively. NCC is the normalized cross-correlation cost function [43]. The relative importance of each term compared to each other are controlled by weights $\gamma_{1}$ and $\gamma_{2}$.

#### 2.3.4. PVSV

The Pulmonary blood Vessel and lobe Surface Varifold (PVSV) algorithm [60] is a shape feature-based (LDDMM) large deformation registration approach that aligns varifold representations of the lung blood vessel skeletons and lobe surfaces. The process for extracting the blood vessel trees and lobe surfaces used for the PVSV algorithm is the same as described in Section 2.2. The skeleton of each branch of the vessel tree was parameterized by a quadratic line segment using a least squares fitting process. The skeletons of the vessel trees and the lobe surfaces were then represented with delta Dirac varifolds using the procedure in [60]. Varifolds were introduced to the field of computational anatomy to overcome the orientation problem of currents [51]. The advantages of matching shape with a varifolds representation are: it does not require pre-knowledge of the point correspondence between shapes, and it can match shapes with large geometric changes.

The PVSV algorithm was chosen to determine whether meaningful biomechanical properties could be extracted using only lung surfaces and vessel tree represented via varifolds. The advantage of using lung surfaces and vessel trees for registration is that it reduces the amount of information needed to represent the lung and that these features are invariant to CT intensity changes caused by breathing. Another advantage of this approach is that it can accurately match surface and vessel structures but it has the disadvantage of needing to interpolate the transformation between these features.

The vessel tree skeletons and lung surfaces are registered as a shape complex with varifolds representation [57], and the overall PVSV cost function is given by

(5) $$C=\gamma_{1}\left|\right|\varphi_{*}C_{m}-C_{f}{\left|\right|}_{W^{\prime }}^{2}+\gamma_{2}\left|\right|\varphi_{*}S_{m}-S_{f}{\left|\right|}_{W^{\prime }}^{2}+\gamma_{3}\int_{0}^{1}||Lv\left(t\right)|{|}^{2}dt,$$

where $C_{m}$ and $C_{f}$ are the blood vessel tree skeletons represented with varifolds, while $S_{m}$ and $S_{f}$ are the lung surfaces with varifolds representation. $\varphi_{*}C_{m}$ is the push forward action of the transformation φ on the varifold $C_{m}$ that transports $C_{m}$ into the space of $C_{f}$, and same for $\varphi_{*}S_{m}$. The relative importance of each term is controlled by the weights $\gamma_{1}$, $\gamma_{2}$ and $\gamma_{3}$.

#### 2.3.5. PLOSL

The PLOSL is a fast unsupervised-learning-based framework developed for 3D pulmonary CT based on population learning (PL) and one-shot learning (OSL) [32]. It uses the same tissue volume preserving and vesselness constraints similarity metrics as the SSTVD method. PLOSL has two training stages: (1) PL which serves as a base model; (2) OSL which generates an individual specific model. The PLOSL algorithm has been shown to produce state-of-the-art deep-learning-based image registration performance [32] with comparable accuracy to traditional iterative image registration algorithms and other deep-learning-based methods for lung CT registration.

The cost function for the PLOSL network consists of three components: SSTVD, SSVMD, and a regularization cost as follows:

(6) $$\begin{array}{c} C=\gamma_{1}\frac{1}{\left|\Omega \right|}\int_{\Omega }{\left(I_{f}\left(x\right)-\left|J_{\varphi^{-1}}\left(x\right)\right|I_{m}\left(\varphi^{-1}\left(x\right)\right)\right)}^{2}dx\\ \;\;\;\;\;\;\;+\gamma_{2}\frac{1}{\left|\Omega \right|}\int_{\Omega }{\left(I_{fvm}\left(x\right)-I_{mvm}\left(\varphi^{-1}\left(x\right)\right)\right)}^{2}dx+\gamma_{3}\int_{\Omega }||\nabla u\left(x\right)|{|}_{L^{2}}^{2}dx, \end{array}$$

where the first two terms are the same as the two terms of the SSTVD method, and the third term is a regularization term and the operator $\nabla =[\frac{\partial }{\partial x_{1}},\frac{\partial }{\partial x_{2}},\frac{\partial }{\partial x_{3}}]$.

### 2.4. Image Registration Parameters

The registration algorithms employed in the study were fundamentally different and therefore, the corresponding parameters and multi-resolution setups were different for each method. All the registration algorithms used a coarse-to-fine multi-resolution framework to prevent themselves from becoming stuck in local minima, speed up computation, and achieve better registration performance. Parameters were determined by optimizing image registration performance by hand using a few typical data sets and hundreds of different parameter combinations for each algorithm. We optimized the algorithms independently to achieve the best performance. The following algorithm-specific parameters were used in this work.

The multi-resolution framework used for the SSTVD algorithm consisted of six image resolutions at 1/8, 1/8, 1/4, 1/4, 1/2 and 1 of the original resolution, respectively. The corresponding B-Spline node spacings were 8, 4, 8, 4, 4 and 4 mm. The cost function described in Equation (1) used weights $\gamma_{1}=\gamma_{2}=1$. The differential operator used was $L=-0.75\nabla^{2}-0.25\nabla \left(\nabla \cdot \right)$.

The multi-resolution framework employed for the GDR algorithm consisted of three image resolutions equal to 1/8, 1/4 and 1/2 of the original resolution, respectively. The corresponding standard deviation of the deformation Gaussian kernel size for the transformation were 60, 30, and 15 mm, respectively. A total of ten time points were used for the large deformation model, and the weight on the image cost $\gamma_{1}=0.06$, and the regularization cost $\gamma_{2}=1$ at each resolution.

The multi-resolution framework used for GSyN algorithm consisted of five image resolutions. The input images were smoothed with a Gaussian kernel with standard deviation 4, 3, 2, 1, and 0 mm and down sampled by a factor of 1/16, 1/8, 1/4, 1/2, and 1, respectively.

The multi-resolution framework used for PVSV algorithm consisted of two resolutions. At the coarse resolution, the shape kernel for the vessel tree skeletons and lung surfaces were set to 5 and 15 mm, respectively. At the fine resolution, the shape kernel for the vessel tree skeletons and lung surfaces were set to 3 and 10 mm, respectively. The corresponding deformation kernel sizes were 40 and 30 mm, respectively. The weights on the vessel tree and surface cost terms were set to $\gamma_{1}=10,000$ and $\gamma_{2}=25$ at coarse resolution, respectively, and to $\gamma_{1}=4$ and $\gamma_{2}=0.015$, respectively, at the fine resolution. The weight on the regularization term is $\gamma_{3}=1$ for the two resolutions.

The population learning U-Net of PLOSL was trained with 2042 subjects randomly selected from the SPIROMICS database. There was no overlap between training data used in PLOSL and the data sets (247 subjects) analyzed in this study. The weights on the terms of the cost function Equation (6) were set to $\gamma_{1}=1$, $\gamma_{2}=1$, and $\gamma_{3}=0.01$, respectively. The number of one-shot iterations was set to 50. The methods were implemented in TensorFlow version 2.3.0 and run on an NVIDIA GeForce GTX 2080Ti GPU with 11 GB of memory. The ADAM optimizer with a learning rate of $10^{-4}$ was used for training.

### 2.5. Image Registration Performance

A total of four measures were used to evaluate image registration performance: (1) lung Lobe Dice Coefficient (LDC); (2) Worst 10% Surface Error (W10SE); (3) Vessel Tree Position Error (VTPE); and (4) Symmetric Closest Skeleton Error (SCSE).

LDC measures the overlap of the moving and fixed lung lobes. The Dice Coefficient (DC) of two overlapping regions $L_{1}$ and $L_{2}$ is given by $DC=\frac{2|L_{1}\cap L_{2}|}{|L_{1}\left|+\right|L_{2}|}$. The human lung has five lobes: right upper lobe, right middle lobe, right lower lobe, left upper lobe, and left lower lobe. The LDC is the average DC of the five lobes.

W10SE is an error measurement of the alignment of two lung surfaces. The W10SE captures the average surface error where two surfaces disagree the most. The process for extracting the lung lobe surface triangulation used for this evaluation is described in Section 2.2. For each vertex *p* of the triangles that parametrize the surface $S_{1}$, its closet point *q* on $S_{2}$ is found, and the distances between *p* and *q* are stored. It is worth noting that *q* is not necessarily a vertex of $S_{2}$ and may instead be on the face of a triangle. As a next step, the distances for all the vertices of $S_{1}$ are sorted from large to small, and the mean of the largest 10% distance is computed, which is denoted as $W10_{S_{1}\to S_{2}}$. Similarly, we compute the mean of worst 10% distance in the opposite direction, i.e., we rank the closest-point distances computed from each vertex of $S_{2}$ to $S_{1}$, then compute the mean of worst 10% again, and denote it as $W10_{S_{2}\to S_{1}}$. The W10SE is defined as: $W10SE=\frac{1}{2}\left(W10_{S_{1}\to S_{2}}+W10_{S_{2}\to S_{1}}\right)$.

VTPE measures the error of the lung blood vessel alignment. The process for extracting the blood vessel tree skeleton used for this evaluation is described in Section 2.2. The closest-point distances from the fixed to the moving segmentation were computed for each voxel in the fixed segmentation and averaged. This process was repeated to compute the average closest-point distance from the moving to the fixed segmentation. The VTPE is defined as the average of these two closest-point distances.

SCSE measures the alignment of the skeletons of the vessel tree. SCSE is similar to VTPE, except the symmetric closest-point distance is computed using the vessel tree skeletons instead of binary vessel segmentations.

### 2.6. Biomechanical Measures

This section briefly summarizes the calculation of *J*, ADI, and SRI [16]. Let F(x) be the deformation gradient tensor at the point *x* in the lung, i.e., the gradient of the transformation from the inspiration-to-expiration CT images at *x*. For each point *x* in the lung, the quantity $F^{T}F$ is the right Cauchy–Green deformation tensor with corresponding eigenvalues $\lambda_{i}^{2}$ such that $\lambda_{1}\geq \lambda_{2}\geq \lambda_{3}$.

The Jacobian determinant J(x) measures the volume change at *x* and can be computed as $J=\lambda_{1}\lambda_{2}\lambda_{3}$. A value of J(x)>1 corresponds to local volume expansion at *x* and a value of J<1 corresponds to local volume contraction at *x*.

ADI measures the magnitude of anisotropy deformation, i.e., it describes how much stretching occurs along one or two directions in a three-dimensional space. The formula for computing ADI is: $\mathrm{ADI}=\sqrt{{\left(\frac{\lambda_{1}-\lambda_{2}}{\lambda_{2}}\right)}^{2}+{\left(\frac{\lambda_{2}-\lambda_{3}}{\lambda_{3}}\right)}^{2}}$. If the volume changes uniformly along each direction (isotropically), then ADI equals zero. The larger the ADI value, the more anisotropic the deformation.

SRI is a measure of the nature of anisotropy, i.e., it measures whether the volume change is predominant along one direction (rod-like) or two directions (slab-like). SRI is computed using the formula: $\mathrm{SRI}=\frac{tan^{-1}\left(\frac{\lambda_{3}\left(\lambda_{1}-\lambda_{2}\right)}{\lambda_{2}\left(\lambda_{2}-\lambda_{3}\right)}\right)}{\pi /2}$. Note that the value of SRI ranges from zero to one where zero corresponds to a slab-like shape and one corresponds to a rod-like shape. SRI is undefined when ADI is zero.

**Figure 2:**
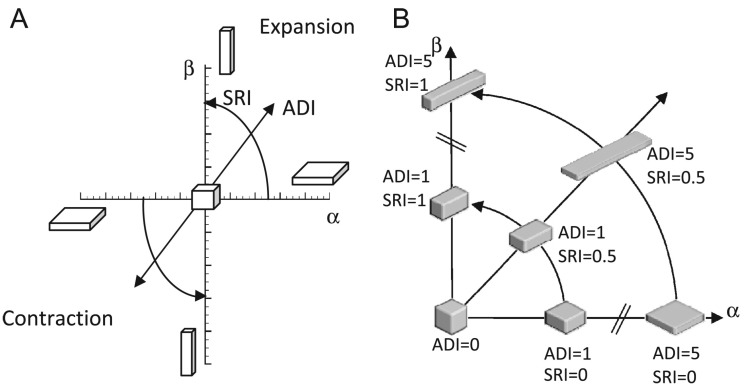
Illustration of regional shape change with respect to ADI and SRI. (**A**) shows the cuboid shape space as a function of ADI and SRI and how these values are related to α and β in terms similar to polar coordinates. The cuboid shape is a uniform cube when ADI = 0. The cuboid shape is flat when SRI = 0 and rod like when SRI = 1. The shape of a uniform cube expands or contracts as the magnitude of ADI increases. (**B**) shows the cuboid shape space for particular values of ADI and SRI. Figure from [16].

Figure 2 illustrates the relationships between ADI and SRI. The α and β axes are defined as following: $\alpha =\frac{J-1}{\left|J-1\right|}\left(\frac{\lambda_{2}}{\lambda_{3}}-1\right)$, and $\beta =\frac{J-1}{\left|J-1\right|}\left(\frac{\lambda_{1}}{\lambda_{2}}-1\right)$ [16]. Notice that the terms in the parentheses for both α and β are always positive due to the relationship between the eigenvalues. Therefore, when *J* is greater than one (i.e., expansion), α and β are both positive (first quadrant of the graph in panel A). Likewise, when *J* is smaller than one (i.e., contraction), then α and β are both negative (third quadrant).

Panel B shows the first quadrant of panel A. This figure illustrates how the ADI and SRI can be thought of as polar coordinates with ADI as the radius and SRI as the angle. Notice that the deformation becomes more anisotropic as ADI increases. At the origin, the ADI=0 corresponding to an isotropic deformation.

## 3. Results

### 3.1. Registration Performance

The performance of the TLC-to-RV image registration was evaluated using registrations computed from 245 unique individuals. The baseline scans of 247 subjects were randomly chosen from the 14 SPIROMICS clinical sites, with 50 subjects from each of GOLD 0, 1, 2, 3 and 47 subjects from GOLD 4. During analysis, one data set was excluded from the GOLD 0 cohort because the RV scan did not cover the entire lung. In addition, one data set was excluded from the GOLD 4 cohort because the RV and TLC scans were nearly identical, i.e., the subject was imaged twice at the same lung volume. The GDR image algorithm failed (i.e., produced a nonsensical correspondence map) on one of each of the GOLD 1, 2, and 3 data sets. The reason for the three GDR image registration failures was due to large shape differences between the TLC and RV scans which caused the GDR to become stuck in a local minimum during the optimization procedure. The measurements from these three registration results were excluded from the analysis.

**Figure 3:**
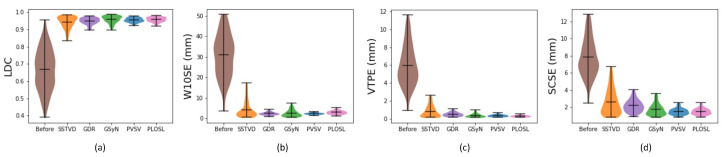
Violin plots of the TLC-to-RV registration performance using (**a**) Lung lobe Dice coefficient (LDC), (**b**) Worst 10% surface error (W10SE), (**c**) Vessel tree position error (VTPE), (**d**) Symmetric closest skeleton error (SCSE). The first column in each graph is the measure before registration. The five remaining columns correspond to values for the five algorithms: Sum-of-Squared-Tissue Volume-Difference (SSTVD), Geodesic Density Regression (GDR), Greedy Symmetric Normalization (GSyN), Pulmonary blood Vessel and lobe Surface Varifold (PVSV) and Population Learning followed by One Shot Learning (PLOSL), respectively. Note that a larger LDC value is better whereas a smaller value is better for the other three evaluation methods.

**Figure A1:**
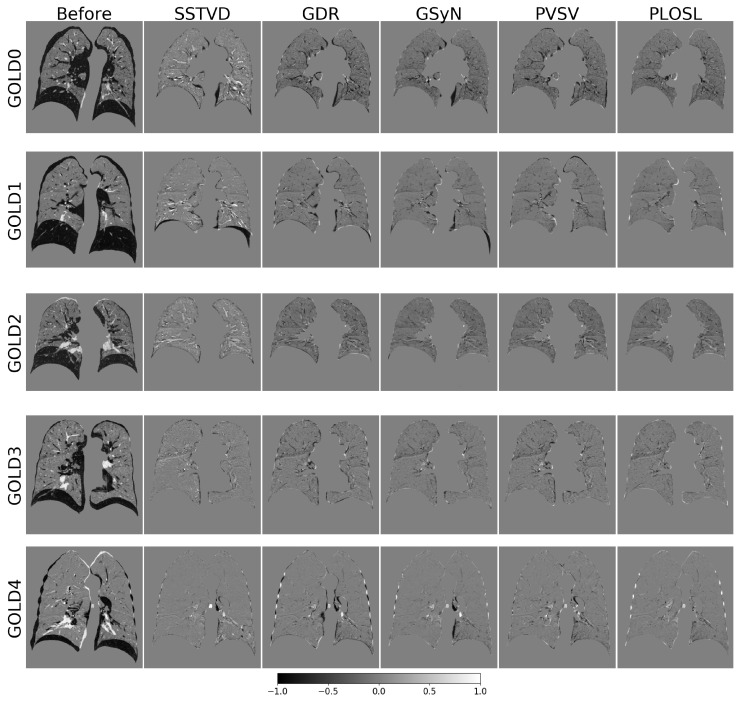
Difference images for the registrations used to compute the Jacobian determinant images shown in Figure 5. The first column shows a 2D coronal cross section of the lungs before registration. The last five images in each row show the same coronal cross section of the lungs that resulted from each registration algorithm. Gray corresponds to a zero difference. Black corresponds to negative values and white corresponds to positive values. A lung mask was used for registration and is the reason why the area outside the lungs is gray.

Figure 3 shows the registration performance for each registration algorithm. The numbers used to generate these plots are summarized in Table 1. The bars at the top, middle, and bottom of the violin plots correspond to the maximum, mean, and minimum of the dataset, respectively. Notice that SSTVD had the largest variance of the five registration methods. This is because the SSTVD method was unable to handle large deformations as well as the other four methods. The performances of GDR and GSyN are similar, and the performances of PVSV and PLOSL are also close and better than the other algorithms. Appendix B shows typical difference images for the five image-registration algorithms across GOLD stage. The results shown in Table 1 and Figure A1 demonstrate that the performance of the five registration algorithms were similar.

### 3.2. Robustness of Inferred Biomechanical Features

**Figure 4:**
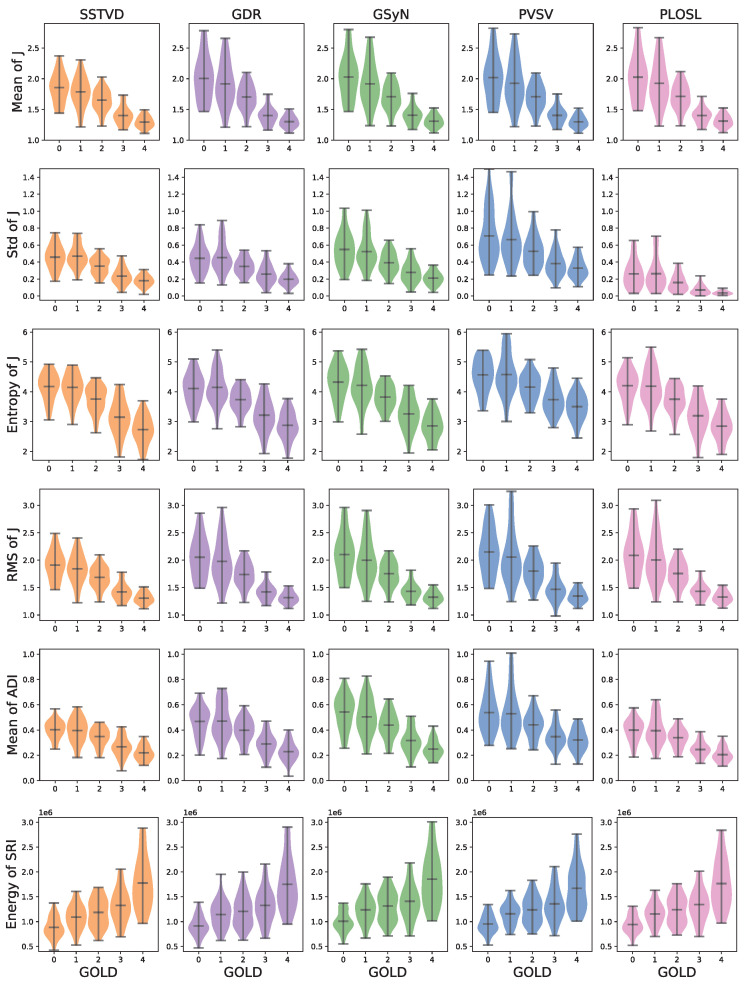
Comparison of biomechanical measures across image registration algorithms as a function of GOLD stages. The measures shown were the only measures to exhibit monotonically increasing/decreasing behavior independent of registration algorithms (GOLD: Global Initiative for Chronic Obstructive Lung Disease, SRI: slab-rod index, ADI: anisotropic deformation index).

Figure 4 shows the mean, standard deviation (std), entropy, and root mean squared (RMS) of *J*, mean of ADI, and entropy of SRI computed with respect to GOLD stage. The numbers used to generate these graphs are summarized in Table A1. This figure shows that these measures increased or decreased monotonically as GOLD increased and that these trends were consistent for all registration algorithms.

**Figure 5:**
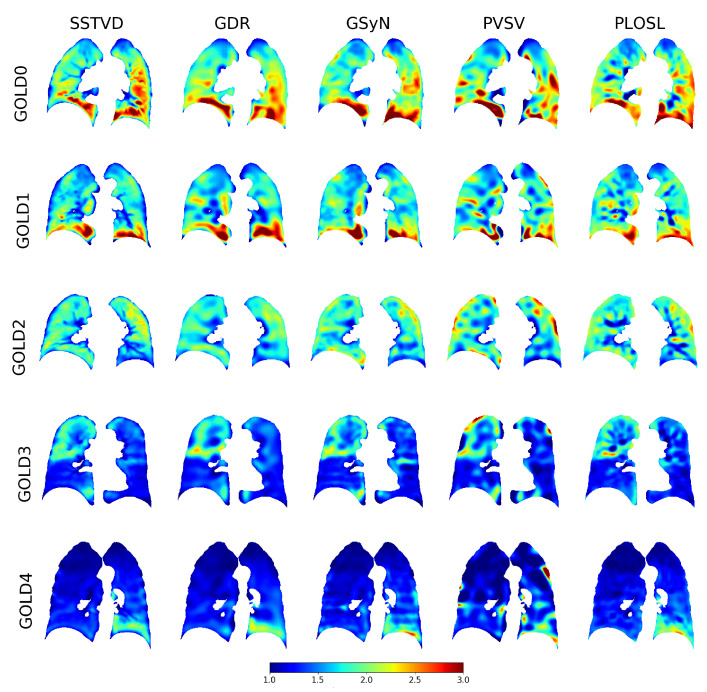
Typical Jacobian determinant images for each GOLD stage and registration algorithm. Dark red regions correspond to regions of large local expansion and dark blue regions correspond to regions with little to no expansion. This figure shows that each algorithm produced similar results for each GOLD stage and that the amount of local lung expansion decreased as the GOLD stage increased.

Figure 5 shows the *J* images for typical subjects selected from each GOLD stage. Each row corresponds to a given subject and each column shows the *J* image computed by a different registration algorithm. Notice that the *J* images in each row (i.e., for a given subject) appear similar to each other, demonstrating some degree of invariance to registration algorithm. Further, note that along each column the mean *J* decreases (i.e., the overall color transitions to darker blue) as GOLD stage increases, regardless of registration algorithm.

**Figure 6:**
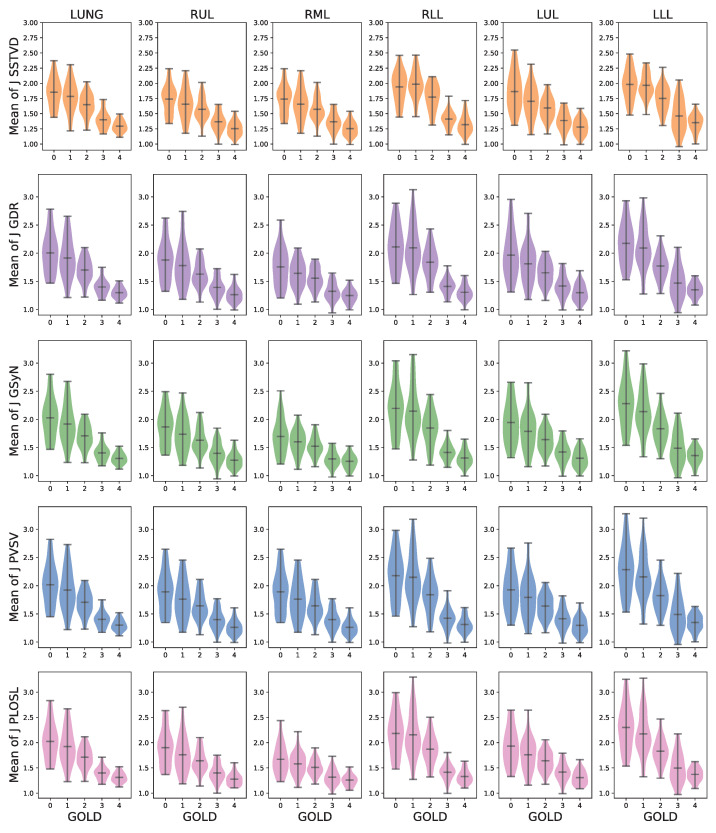
The robustness of average *J* with respect to different registration algorithms gloablly and on a lobe-by-lobe basis. For each row, the first column is the averaged *J* of lung region (LUNG). The five remaining columns correspond to the averaged results within five lobes of the lung, respectively. RUL, RML, and RLL refer to right upper, middle and lower lobe, respectively, whereas LUL and LLL represent left upper and lower lobe. Each row corresponds to the five registration algorithms: SSTVD, GDR, GSyN, PVSV and PLOSL, respectively.

Figure 6 shows the distribution of the average *J* computed within the whole lung and each lobe for each registration algorithm. The numbers used to generate these graphs are summarized in Table A2. These plots show that the average Jacobian determinant decreased as the GOLD stage increased globally, on a lobe-by-lobe basis, and that these trends held for all the registration algorithms.

**Figure 7:**
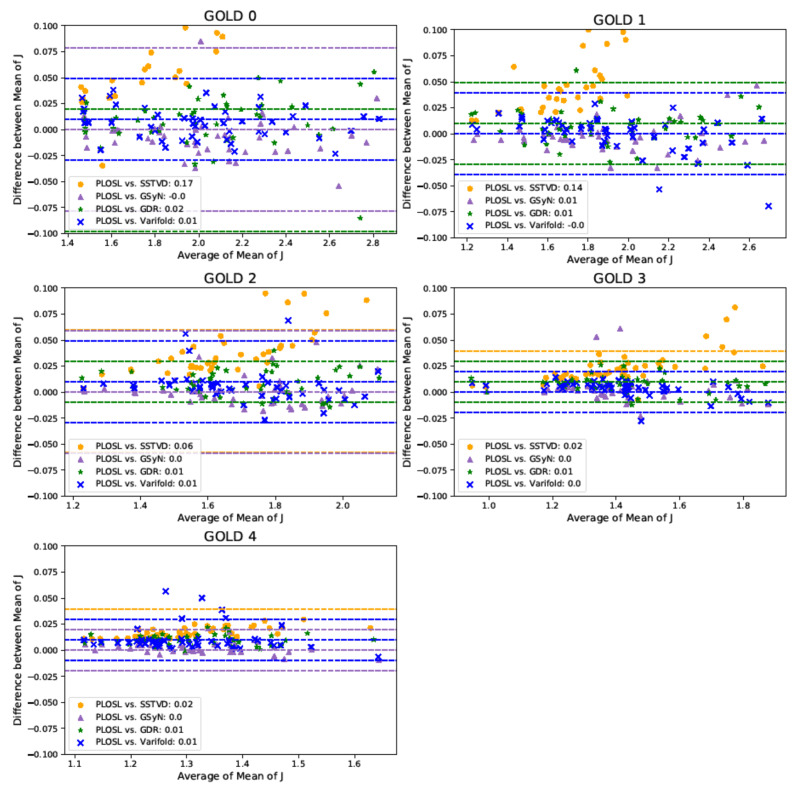
Multiple Comparison Bland–Altman Plots of Mean Jacobian. The shapes and colors in the legend show the Bland–Altman plot for a given algorithm comparison. There are three dashed lines for each comparison corresponding to the average difference and to the 95% limits of agreement, i.e., the average difference ±1.96 standard deviation (SD) of the difference. The color of the dashed lines correspond to the comparison denoted in the legend. Some of the dashed lines may appear to be missing, but that is because they are covered up by other dashed lines or out of range of the *y*-axis.

Figure 7 shows Bland–Altman plots of the mean *J* and compares the measurements generated by all five image-registration algorithms. These Bland–Altman plots show the degree of agreement between the measurement methods on an individual basis and support the results shown in the violin plots (see Figure 3, Figure 4 and Figure 6). Each graph shows four Bland–Altman plots corresponding to PLOSL vs. SSTVD, PLOSL vs. GSyN, PLOSL vs. GDR, and PLOSL vs. PVSV, respectively. Note that the horizontal axis is different for each GOLD stage plot since the average amount of lung deformation from expiration to inspiration decreased as GOLD stage increased. The downward trend in the GOLD 0 plot as the average *J* increases implies that the GSyN, GDR, PVSV, and PLOSL methods all diverged from the SSTVD method for subjects that had more RV-TLC expansion. A similar downward trend is evident in the GOLD 1 plot. The GOLD 2, 3, and 4 plots show less disagreement between the five registration methods. This can be explained by the fact that there was much less RV-TLC expansion (note the ranges on the horizontal axes) for GOLD 2, 3, and 4 subjects compared to GOLD 0 and 1 subjects. These plots further confirm the consistent trends detected by the five registration algorithms that the average *J* decreases as GOLD stage increases (as shown in the *x*-axis).

## 4. Discussion and Conclusions

This paper investigated the effects of using different image registration algorithms to extract lung biomechanical features from inspiration and expiration CT images of subjects with COPD. In this work, we evaluated both small deformation (SSTVD and PLOSL) and large deformation (GDR, GSyN, and PVSV) image registration algorithms. The two main differences between large and small transformation models are that a large transformation model allows for curved particle paths from the moving image to target image, and large transformation models guarantee a one-to-one correspondence between the moving and target images.

Figure 6 shows that the average *J* of the SSTVD method was smaller than those of the other four registration algorithms. One explanation for this is that the regularization used for the SSTVD algorithm produced smoother deformations than the other approaches and was therefore unable to accommodate as much local deformation. In addition, these results demonstrate that a deep-learning-based image registration method (PLOSL) can have similar average performance to traditional iterative image registration algorithms (GDR, GSyN, and PVSV).

In total, five biomechanical measures (mean, standard deviation, and entropy of *J*, mean of ADI, and energy of SRI) were shown to follow a statistically significant monotonically increasing or decreasing trend for the entire lung and on a lobe-by-lobe basis as GOLD stage increased. These trends held for all five registration algorithms. This study showed that the expansion between RV and TLC decreases as GOLD stage increases in the whole lung and within each lobe of the lung, which may potentially allow for detection, monitoring, and regional evaluation of COPD. These findings support the work of Ding et al. [71], who showed that lung function and mechanics vary regionally on a lobe-by-lobe basis. This work also complements the work by Bhatt et al. [3], who reported spatial correlations between regions of functional decline identified by the Jacobian determinant and regions of emphysematous lung tissue.

The SSTVD, GDR, and PLOSL algorithms employed in this study were implemented by our group. The GSyN used was from the ANTs [70] and PVSV registration results used the Deformetrica [72] software version 3.0.

The SSTVD, GDR, GSyN, and PVSV algorithms were run on a single high-memory Argon Phase 1 compute node on the University of Iowa High-Performance Argon Cluster (https://hpc.uiowa.edu/ (accessed on 10 October 2022)). The node has two Xeon E5-2680v4 (28 Cores at 2.4 GHz) for a total of 56 compute slots, 512 GB RAM, 1 Gbps Ethernet, 100 Gbps Omnipath, and HPL benchmark performance of 766.1 GFlops, and cost approximately USD 7500. The PLOSL algorithm was run on an NVIDIA Tesla 2080Ti GPU. The computation time for registration varied depending on the amount of deformation required. In general, GOLD 4 cases demonstrated smaller shape change between inspiration and expiration compared to GOLD 0 cases. On average, the SSTVD, GDR, GSyN, and PVSV took an hour to register a 3D pair of images. The GDR algorithm required the most computational resources and took almost 2 h for registrations that required extensive deformation. In contrast, the deep learning PLOSL algorithm was 1.5 orders of magnitude faster than the other four registration methods. Once trained, the PLOSL algorithm took approximately 1.25 min to register a 3D pair of images. Thus, for this study, the PLOSL algorithm seems to be the preferred registration algorithm due to its faster run time and comparable performance. The only caveat to using the PLOSL algorithm is that it needed a large number of data sets for training. In the absence of training data, one of the other registration algorithms should be selected.

The SSTVD and GDR both used the same loss function. However, these methods differ in how the transformations are parameterized. The loss function defines what features correspond for matching while the transformation parameterization defines the amount of deformation that is allowed when matching. The SSTVD transformation is parameterized by small deformation B-splines whereas GDR transformation is parameterized by a high-dimensional temporal and spatially varying velocity vector field. The result of this is that the GDR algorithm has orders of magnitude more degrees of freedom to match two image volumes compared to the SSTVD algorithm. Having more degrees of freedom is not always better if they are not needed. This paper shows that both algorithms performed similarly for the current task of registering inspiration-to-expiration CT lung volumes. Thus, one can conclude that the extra degrees of freedom, extra computation time and additional computational resources for this task were not needed.

The results presented in this paper demonstrate that five different and varied registration methods can all produce similar measures of lung biomechanics. Such biomechanical measures, when analyzed on a local level, further strengthen the link between spirometry and quantitative CT and may help explain the functional changes missed using spirometry or CT density thresholds alone [3]. There are several current and potential implications of our work. Existing measures of lung function and structure are limited by the lack of spatial information and by the lack of sensitivity to detect early changes. These image-registration-based biomechanical measures are robust in detecting early abnormalities that have been linked to important clinical outcomes, including respiratory quality of life, functional capacity, and all-cause mortality [21]. Spatial information on a lobar and sub-lobar level are increasingly being recognized as important in patient selection for interventional COPD therapies such as bronchoscopic and surgical lung volume reduction. These measures may provide additional information, beyond density-based measures, about the health of the target and ipsilateral non-target lobes for such procedures. Biomechanical measures may also help inform patient and target selection for radiation therapy and for lobar and segmental lung resection in lung cancer. Using spirometry, CT density measures, and biomechanical features together may help provide mechanistic insights to explain changes in lung function in COPD and related lung diseases.

## Figures and Tables

**Table 1:** Comparison of registration performance of inspiration–expiration CT registration between four iterative-registration algorithms and a deep-learning-based registration method. Results are provided in mean ± standard deviation format.

Method	LDC	W10SE (mm)	VTPE (mm)	SCSE (mm)
Before	0.67 ± 0.12	30.90 ± 10.18	5.97 ± 2.17	7.83 ± 2.11
SSTVD	0.94 ± 0.03	4.21 ± 3.97	0.82 ± 0.64	2.61 ± 1.45
GDR	0.95 ± 0.02	2.40 ± 0.67	0.51 ± 0.20	2.18 ± 0.68
GSyN	0.96 ± 0.02	2.54 ± 1.62	0.37 ± 0.18	1.72 ± 0.65
PVSV	0.96 ± 0.01	2.31 ± 0.36	0.35 ± 0.11	1.51 ± 0.35
PLOSL	0.96 ± 0.01	2.93 ± 0.79	0.30 ± 0.08	1.50 ± 0.34

## Data Availability

The SPIROMICS data are available at www.spiromics.org (accessed on 10 October 2022).

## Appendix A. Quantitative Results for Figure

**Table A1:** Quantitative Results for Figure 4. Comparison of biomechanical measures across image registration algorithms as a function of GOLD stages. Results are provided in mean ± standard deviation format.

Biomechanical Measure	Method	GOLD 0	GOLD 1	GOLD 2	GOLD 3	GOLD 4
Mean of *J*	SSTVD	1.85 ± 0.24	1.78 ± 0.24	1.65 ± 0.17	1.40 ± 0.14	1.29 ± 0.09
	GDR	2.01 ± 0.36	1.92 ± 0.35	1.70 ± 0.20	1.40 ± 0.14	1.30 ± 0.09
	GSyN	2.03 ± 0.35	1.92 ± 0.35	1.71 ± 0.20	1.40 ± 0.14	1.31 ± 0.10
	PVSV	2.02 ± 0.36	1.93 ± 0.36	1.71 ± 0.21	1.40 ± 0.14	1.30 ± 0.09
	PLOSL	2.03 ± 0.36	1.92 ± 0.35	1.71 ± 0.20	1.40 ± 0.12	1.31 ± 0.09
Std of *J*	SSTVD	0.46 ± 0.13	0.47 ± 0.13	0.35 ± 0.10	0.23 ± 0.10	0.18 ± 0.06
	GDR	0.44 ± 0.16	0.45 ± 0.17	0.35 ± 0.10	0.26 ± 0.11	0.20 ± 0.07
	GSyN	0.55 ± 0.20	0.52 ± 0.19	0.39 ± 0.12	0.28 ± 0.12	0.21 ± 0.07
	PVSV	0.71 ± 0.29	0.66 ± 0.29	0.53 ± 0.18	0.38 ± 0.15	0.33 ± 0.11
	PLOSL	0.26 ± 0.16	0.26 ± 0.19	0.16 ± 0.10	0.07 ± 0.05	0.04 ± 0.02
Entropy of *J*	SSTVD	4.17 ± 0.45	4.15 ± 0.46	3.75 ± 0.43	3.15 ± 0.56	2.73 ± 0.45
	GDR	4.11 ± 0.50	4.14 ± 0.54	3.73 ± 0.38	3.21 ± 0.54	2.88 ± 0.45
	GSyN	4.32 ± 0.57	4.21 ± 0.63	3.82 ± 0.39	3.26 ± 0.53	2.85 ± 0.43
	PVSV	4.57 ± 0.53	4.58 ± 0.68	4.16 ± 0.43	3.73 ± 0.46	3.50 ± 0.43
	PLOSL	4.21 ± 0.50	4.18 ± 0.59	3.75 ± 0.42	3.20 ± 0.56	2.85 ± 0.45
RMS of *J*	SSTVD	1.91 ± 0.26	1.84 ± 0.27	1.69 ± 0.19	1.42 ± 0.16	1.31 ± 0.10
	GDR	2.05 ± 0.36	1.98 ± 0.39	1.74 ± 0.22	1.42 ± 0.15	1.32 ± 0.10
	GSyN	2.10 ± 0.39	2.00 ± 0.39	1.75 ± 0.22	1.43 ± 0.15	1.33 ± 0.10
	PVSV	2.15 ± 0.41	2.06 ± 0.47	1.80 ± 0.25	1.47 ± 0.22	1.35 ± 0.12
	PLOSL	2.09 ± 0.38	2.01 ± 0.41	1.76 ± 0.22	1.43 ± 0.15	1.33 ± 0.10
Mean of ADI	SSTVD	0.40 ± 0.07	0.40 ± 0.09	0.35 ± 0.07	0.27 ± 0.08	0.22 ± 0.06
	GDR	0.47 ± 0.11	0.47 ± 0.14	0.40 ± 0.09	0.29 ± 0.08	0.23 ± 0.07
	GSyN	0.54 ± 0.13	0.50 ± 0.14	0.44 ± 0.10	0.32 ± 0.10	0.25 ± 0.07
	PVSV	0.54 ± 0.16	0.53 ± 0.19	0.44 ± 0.10	0.35 ± 0.09	0.32 ± 0.08
	PLOSL	0.40 ± 0.08	0.39 ± 0.12	0.34 ± 0.07	0.25 ± 0.06	0.21 ± 0.06
Energy of SRI ($\times 10^{6}$)	SSTVD	0.88 ± 0.20	1.09 ± 0.24	1.19 ± 0.28	1.32 ± 0.33	1.77 ± 0.50
	GDR	0.92 ± 0.19	1.14 ± 0.27	1.21 ± 0.30	1.32 ± 0.33	1.75 ± 0.51
	GSyN	1.01 ± 0.20	1.24 ± 0.25	1.31 ± 0.29	1.41 ± 0.34	1.85 ± 0.52
	PVSV	0.96 ± 0.18	1.16 ± 0.21	1.24 ± 0.26	1.35 ± 0.32	1.67 ± 0.44
	PLOSL	0.94 ± 0.18	1.16 ± 0.22	1.24 ± 0.27	1.34 ± 0.32	1.76 ± 0.48

## Appendix B. Sample Difference Images

Figure A1 shows difference images associated with the registrations used to compute the Jacobian images shown in Figure 5. This figure shows typical difference images for all five image registration algorithms across GOLD stages. All five image-registration algorithms performed equally well with respect to difference images except for three failures of the GDR algorithm. The GDR failed because it contained too many degrees of freedom and became stuck in local minima. The lungs were segmented from the original CT before registration. The most obvious registration errors occurred near the lung mask boundaries. Subtle correspondence errors in the lung parenchyma are difficult to observe in these difference images since a zero gray-scale intensity does not imply correct correspondence.

## Appendix C. Quantitative Results for Figure 6

**Table A2:** Quantitative Results for Figure 6. Comparison of average *J* across image registration algorithms as a function of GOLD stages on both a global and lobe-by-lobe basis. Results are provided in mean ± standard deviation format.

Method	Region	GOLD 0	GOLD 1	GOLD 2	GOLD 3	GOLD 4
SSTVD	LUNG	1.85 ± 0.24	1.78 ± 0.24	1.65 ± 0.17	1.40 ± 0.14	1.29 ± 0.09
	RUL	1.74 ± 0.23	1.66 ± 0.25	1.57 ± 0.18	1.37 ± 0.15	1.25 ± 0.12
	RML	1.74 ± 0.23	1.66 ± 0.25	1.57 ± 0.18	1.37 ± 0.15	1.25 ± 0.12
	RLL	1.94 ± 0.25	1.98 ± 0.22	1.77 ± 0.22	1.41 ± 0.13	1.32 ± 0.15
	LUL	1.87 ± 0.31	1.70 ± 0.26	1.59 ± 0.17	1.39 ± 0.16	1.28 ± 0.13
	LLL	1.98 ± 0.25	1.97 ± 0.19	1.75 ± 0.20	1.46 ± 0.26	1.35 ± 0.14
GDR	LUNG	2.01 ± 0.36	1.92 ± 0.35	1.70 ± 0.20	1.40 ± 0.14	1.30 ± 0.09
	RUL	1.88 ± 0.35	1.78 ± 0.37	1.63 ± 0.21	1.39 ± 0.17	1.26 ± 0.13
	RML	1.76 ± 0.33	1.64 ± 0.23	1.56 ± 0.18	1.32 ± 0.16	1.25 ± 0.12
	RLL	2.11 ± 0.38	2.09 ± 0.40	1.84 ± 0.27	1.41 ± 0.14	1.30 ± 0.13
	LUL	1.97 ± 0.40	1.81 ± 0.34	1.65 ± 0.20	1.42 ± 0.18	1.30 ± 0.15
	LLL	2.18 ± 0.36	2.09 ± 0.37	1.77 ± 0.22	1.47 ± 0.28	1.35 ± 0.13
GSyN	LUNG	2.03 ± 0.35	1.92 ± 0.35	1.71 ± 0.20	1.40 ± 0.14	1.31 ± 0.10
	RUL	1.86 ± 0.31	1.73 ± 0.31	1.63 ± 0.22	1.40 ± 0.19	1.28 ± 0.14
	RML	1.70 ± 0.30	1.60 ± 0.21	1.52 ± 0.16	1.30 ± 0.14	1.25 ± 0.12
	RLL	2.20 ± 0.42	2.15 ± 0.43	1.85 ± 0.30	1.41 ± 0.14	1.31 ± 0.14
	LUL	1.94 ± 0.36	1.79 ± 0.33	1.64 ± 0.20	1.42 ± 0.17	1.31 ± 0.15
	LLL	2.28 ± 0.41	2.14 ± 0.38	1.83 ± 0.25	1.49 ± 0.29	1.36 ± 0.15
PVSV	LUNG	2.02 ± 0.36	1.93 ± 0.36	1.71 ± 0.21	1.40 ± 0.14	1.30 ± 0.09
	RUL	1.89 ± 0.35	1.76 ± 0.34	1.64 ± 0.23	1.40 ± 0.17	1.26 ± 0.13
	RML	1.89 ± 0.35	1.76 ± 0.34	1.64 ± 0.23	1.40 ± 0.17	1.26 ± 0.13
	RLL	2.18 ± 0.41	2.15 ± 0.43	1.84 ± 0.30	1.42 ± 0.17	1.31 ± 0.14
	LUL	1.93 ± 0.37	1.79 ± 0.35	1.64 ± 0.20	1.42 ± 0.18	1.30 ± 0.15
	LLL	2.28 ± 0.43	2.16 ± 0.40	1.83 ± 0.26	1.49 ± 0.30	1.35 ± 0.15
PLOSL	LUNG	2.03 ± 0.36	1.92 ± 0.35	1.71 ± 0.20	1.40 ± 0.12	1.31 ± 0.09
	RUL	1.90 ± 0.36	1.76 ± 0.35	1.64 ± 0.22	1.40 ± 0.17	1.28 ± 0.12
	RML	1.67 ± 0.29	1.58 ± 0.22	1.51 ± 0.15	1.31 ± 0.15	1.26 ± 0.11
	RLL	2.18 ± 0.41	2.16 ± 0.43	1.87 ± 0.28	1.41 ± 0.15	1.33 ± 0.13
	LUL	1.93 ± 0.36	1.76 ± 0.31	1.64 ± 0.20	1.42 ± 0.17	1.31 ± 0.14
	LLL	2.30 ± 0.43	2.17 ± 0.41	1.83 ± 0.25	1.50 ± 0.29	1.37 ± 0.13

## References

[1] Celli B.R., Wedzicha J.A. (2019). Update on clinical aspects of chronic obstructive pulmonary disease. N. Engl. J. Med..

[2] Soriano J.B., Abajobir A.A., Abate K.H., Abera S.F., Agrawal A., Ahmed M.B., Aichour A.N., Aichour I., Aichour M.T.E., Alam K. (2017). Global, regional, and national deaths, prevalence, disability-adjusted life years, and years lived with disability for chronic obstructive pulmonary disease and asthma, 1990–2015: A systematic analysis for the Global Burden of Disease Study 2015. Lancet Respir. Med..

[3] Bhatt S.P., Bodduluri S., Hoffman E.A., Newell J.D., Sieren J.C., Dransfield M.T., Reinhardt J.M. (2017). Computed Tomography Measure of Lung at Risk and Lung Function Decline in Chronic Obstructive Pulmonary Disease. Am. J. Respir. Crit. Care Med..

[4] Corhay J.L., Dang D.N., Van Cauwenberge H., Louis R. (2014). Pulmonary Rehabilitation and COPD: Providing Patients a Good Environment for Optimizing Therapy. Int. J. Chronic Obstr. Pulm. Dis..

[5] Vestbo J., Hurd S.S., Agustí A.G., Jones P.W., Vogelmeier C., Anzueto A., Barnes P.J., Fabbri L.M., Martinez F.J., Nishimura M. (2013). Global Strategy for the Diagnosis, Management, and Prevention of Chronic Obstructive Pulmonary Disease: GOLD Executive Summary. Am. J. Respir. Crit. Care Med..

[6] Couper D., LaVange L.M., Han M., Barr R.G., Bleecker E., Hoffman E.A., Kanner R., Kleerup E., Martinez F.J., Woodruff P.G. (2014). Design of the Subpopulations and Intermediate Outcomes in COPD Study (SPIROMICS). Thorax.

[7] Regan E.A., Hokanson J.E., Murphy J.R., Make B., Lynch D.A., Beaty T.H., Curran-Everett D., Silverman E.K., Crapo J.D. (2011). Genetic Epidemiology of COPD (COPDGene) Study Design. COPD J. Chronic Obstr. Pulm. Dis..

[8] Hoffman E.A., Jiang R., Baumhauer H., Brooks M.A., Carr J.J., Detrano R., Reinhardt J., Rodriguez J., Stukovsky K., Wong N.D. (2009). Reproducibility and Validity of Lung Density Measures from Cardiac CT Scans—The Multi-Ethnic Study of Atherosclerosis (MESA) Lung Study. Acad. Radiol..

[9] Shaker S., Dirksen A., Laursen L.C., Skovgaard L., Holstein-Rathlou N.H. (2004). Volume Adjustment of Lung Density by Computed Tomography Scans in Patients with Emphysema. Acta Radiol..

[10] Stoel B.C., Bakker M.E., Stolk J., Dirksen A., Stockley R.A., Piitulainen E., Russi E.W., Reiber J.H. (2004). Comparison of the Sensitivities of 5 Different Computed Tomography Scanners for the Assessment of the Progression of Pulmonary Emphysema: A Phantom Study. Investig. Radiol..

[11] Sørensen L., Nielsen M., Lo P., Ashraf H., Pedersen J.H., De Bruijne M. (2011). Texture-based Analysis of COPD: A Data-Driven Approach. IEEE Trans. Med. Imaging.

[12] Sorensen L., Shaker S.B., De Bruijne M. (2010). Quantitative Analysis of Pulmonary Emphysema Using Local Binary Patterns. IEEE Trans. Med. Imaging.

[13] Ungprasert P., Wilton K.M., Ernste F.C., Kalra S., Crowson C.S., Rajagopalan S., Bartholmai B.J. (2017). Novel Assessment of Interstitial Lung Disease Using the “Computer-Aided Lung Informatics for Pathology Evaluation and Rating” (CALIPER) Software System in Idiopathic Inflammatory Myopathies. Lung.

[14] Hasenstab K.A., Yuan N., Retson T., Conrad D.J., Kligerman S., Lynch D.A., Hsiao A., Investigators C. (2021). Automated CT Staging of Chronic Obstructive Pulmonary Disease Severity for Predicting Disease Progression and Mortality with a Deep Learning Convolutional Neural Network. Radiol. Cardiothorac. Imaging.

[15] Galbán C.J., Han M.K., Boes J.L., Chughtai K.A., Meyer C.R., Johnson T.D., Galbán S., Rehemtulla A., Kazerooni E.A., Martinez F.J. (2012). Computed Tomography—Based Biomarker Provides Unique Signature for Diagnosis of COPD Phenotypes and Disease Progression. Nat. Med..

[16] Amelon R., Cao K., Ding K., Christensen G.E., Reinhardt J.M., Raghavan M.L. (2011). Three-Dimensional Characterization of Regional Lung Deformation. J. Biomech..

[17] Bodduluri S., Newell J.D., Hoffman E.A., Reinhardt J.M. (2013). Registration-based Lung Mechanical Analysis of Chronic Obstructive Pulmonary Disease (COPD) using a Supervised Machine Learning Framework. Acad. Radiol..

[18] Müller N.L., Staples C.A., Miller R.R., Abboud R.T. (1988). “Density mask”: An objective method to quantitate emphysema using computed tomography. Chest.

[19] Newman K.B., Lynch D.A., Newman L.S., Ellegood D., Newell Jr J.D. (1994). Quantitative computed tomography detects air trapping due to asthma. Chest.

[20] Bhatt S.P., Bodduluri S., Newell J.D., Hoffman E.A., Sieren J.C., Han M.K., Dransfield M.T., Reinhardt J.M., Investigators C. (2016). CT-derived biomechanical metrics improve agreement between spirometry and emphysema. Acad. Radiol..

[21] Bodduluri S., Bhatt S.P., Hoffman E.A., Newell J.D., Martinez C.H., Dransfield M.T., Han M.K., Reinhardt J.M. (2017). Biomechanical CT metrics are associated with patient outcomes in COPD. Thorax.

[22] Pan Y., Christensen G.E., Durumeric O.C., Sarah E., Gerard S.P.B., Barr R.G., Hoffman E.A., Reinhardt J.M. Assessment Of Lung Biomechanics In COPD Using Image Registration. Proceedings of the 2020 IEEE 17th International Symposium on Biomedical Imaging (ISBI 2020).

[23] Chaudhary M.F., Pan Y., Wang D., Bodduluri S., Bhatt S.P., Comellas A.P., Hoffman E.A., Christensen G.E., Reinhardt J.M. (2020). Registration-Invariant Biomechanical Features for Disease Staging of COPD in SPIROMICS. Proceedings of the International Workshop on Thoracic Image Analysis.

[24] Vishnevskiy V., Gass T., Szekely G., Tanner C., Goksel O. (2016). Isotropic total variation regularization of displacements in parametric image registration. IEEE Trans. Med. Imaging.

[25] Rühaak J., Polzin T., Heldmann S., Simpson I.J., Handels H., Modersitzki J., Heinrich M.P. (2017). Estimation of large motion in lung CT by integrating regularized keypoint correspondences into dense deformable registration. IEEE Trans. Med. Imaging.

[26] Heinrich M.P., Jenkinson M., Brady M., Schnabel J.A. (2013). MRF-based deformable registration and ventilation estimation of lung CT. IEEE Trans. Med. Imaging.

[27] Yang X., Kwitt R., Styner M., Niethammer M. (2017). Quicksilver: Fast predictive image registration—A deep learning approach. Neuroimage.

[28] Eppenhof K.A.J., Pluim J.P.W. (2019). Pulmonary CT Registration Through Supervised Learning With Convolutional Neural Networks. IEEE Trans. Med. Imaging.

[29] Balakrishnan G., Zhao A., Sabuncu M.R., Guttag J., Dalca A.V. (2019). VoxelMorph: A Learning Framework for Deformable Medical Image Registration. IEEE Trans. Med. Imaging.

[30] Jiang Z., Yin F.F., Ge Y., Ren L. (2020). A multi-scale framework with unsupervised joint training of convolutional neural networks for pulmonary deformable image registration. Phys. Med. Biol..

[31] Fu Y., Lei Y., Wang T., Higgins K., Bradley J.D., Curran W.J., Liu T., Yang X. (2020). LungRegNet: An unsupervised deformable image registration method for 4D-CT lung. Med. Phys..

[32] Wang D., Pan Y., Durumeric O.C., Reinhardt J.M., Hoffman E.A., Schroeder J.D., Christensen G.E. (2022). PLOSL: Population Learning Followed by One Shot Learning Pulmonary Image Registration Using Tissue Volume Preserving and Vesselness Constraints. Med Image Anal..

[33] Sieren J.P., Newell Jr J.D., Barr R.G., Bleecker E.R., Burnette N., Carretta E.E., Couper D., Goldin J., Guo J., Han M.K. (2016). SPIROMICS Protocol for Multicenter Quantitative Computed Tomography to Phenotype the Lungs. Am. J. Respir. Crit. Care Med..

[34] Gerard S.E., Herrmann J., Kaczka D.W., Musch G., Fernandez-Bustamante A., Reinhardt J.M. (2020). Multi-resolution convolutional neural networks for fully automated segmentation of acutely injured lungs in multiple species. Med Image Anal..

[35] Gerard S.E., Reinhardt J.M. (2019). Pulmonary Lobe Segmentation Using A Sequence of Convolutional Neural Networks For Marginal Learning. Proceedings of the 2019 IEEE 16th International Symposium on Biomedical Imaging (ISBI 2019).

[36] Gerard S.E., Patton T.J., Christensen G.E., Bayouth J.E., Reinhardt J.M. (2018). FissureNet: A deep Learning Approach for Pulmonary Fissure Detection in CT Images. IEEE Trans. Med. Imaging.

[37] Sieger D., Botsch M. (2011). Design, Implementation, and Evaluation of the Surface_Mesh Data Structure. Proceedings of the 20th International Meshing Roundtable.

[38] Cohen-Steiner D., Alliez P., Desbrun M. (2004). Variational Shape Approximation. [Research Report] RR-5371, INRIA. https://hal.archives-ouvertes.fr/inria-00070632.

[39] Jerman T., Pernuš F., Likar B., Špiclin Ž. Beyond Frangi: An improved multiscale vesselness filter. Proceedings of the SPIE Medical Imaging. Image Processing; Volume 94132A; International Society for Optics and Photonics.

[40] Homann H. (2007). Implementation of a 3D thinning algorithm. Insight J..

[41] Beg M.F., Miller M.I., Trouvé A., Younes L. (2005). Computing Large Deformation Metric Mappings via Geodesic Flows of Diffeomorphisms. Int. J. Comput. Vis..

[42] Christensen G.E., Johnson H.J. (2001). Consistent image registration. IEEE Trans. Med. Imaging.

[43] Song G., Tustison N., Avants B., Gee J.C. (2010). Lung CT Image Registration using Diffeomorphic Transformation Models. Medical Image Analysis for the Clinic: A Grand Challenge.

[44] Pluim J.P., Maintz J.A., Viergever M.A. (2003). Mutual-information-based registration of medical images: A survey. IEEE Trans. Med. Imaging.

[45] Cao K., Du K., Ding K., Reinhardt J.M., Christensen G.E. (2010). Regularized Nonrigid Registration of Lung CT Images by Preserving Tissue Volume and Vesselness Measure. Grand Challenges Med. Image Anal..

[46] Yin Y., Hoffman E.A., Lin C.L. (2009). Mass Preserving Non-Rigid Registration of CT Lung Images Using Cubic B-spline. Med. Phys..

[47] Gorbunova V., Sporring J., Lo P., Loeve M., Tiddens H.A., Nielsen M., Dirksen A., de Bruijne M. (2012). Mass Preserving Image Registration for Lung CT. Med. Image Anal..

[48] Guy C.L., Weiss E., Christensen G.E., Jan N., Hugo G.D. (2018). CALIPER: A deformable Image Registration Algorithm for Large Geometric Changes during Radiotherapy for Locally-Advanced Non-Small Cell Lung Cancer. Med. Phys..

[49] Besl P.J., McKay N.D. (1992). Method for Registration of 3-D Shapes. Proceedings of the Sensor fusion IV: Control Paradigms and Data Structures. International Society for Optics and Photonics.

[50] Liu Y. (2004). Improving ICP with easy implementation for free-form surface matching. Pattern Recognit..

[51] Charon N., Trouvé A. (2013). The Varifold Representation of Nonoriented Shapes for Diffeomorphic Registration. SIAM J. Imaging Sci..

[52] Durrleman S., Pennec X., Trouvé A., Thompson P., Ayache N. (2008). Inferring Brain Variability from Diffeomorphic Deformations of Currents: An Integrative Approach. Med. Image Anal..

[53] Durrleman S., Pennec X., Trouvé A., Ayache N. (2008). Sparse Approximation of Currents for Statistics on Curves and Surfaces. Medical Image Computing and Computer-Assisted Intervention–MICCAI 2008.

[54] Durrleman S. (2010). Statistical Models of Currents for Measuring the Variability of Anatomical Curves, Surfaces and their Evolution. Ph.D. Thesis.

[55] Durrleman S., Prastawa M., Gerig G., Joshi S. (2011). Optimal Data-Driven Sparse Parameterization of Diffeomorphisms for Population Analysis. Proceedings of the Biennial International Conference on Information Processing in Medical Imaging.

[56] Durrleman S., Allassonnière S., Joshi S. (2013). Sparse Adaptive Parameterization of Variability in Image Ensembles. Int. J. Comput. Vis..

[57] Durrleman S., Prastawa M., Charon N., Korenberg J.R., Joshi S., Gerig G., Trouvé A. (2014). Morphometry of Anatomical Shape Complexes with Dense Deformations and Sparse Parameters. NeuroImage.

[58] Gorbunova V., Durrleman S., Lo P., Pennec X., De Bruijne M. (2010). Lung CT Registration Combining Intensity, Curves and Surfaces. Proceedings of the 2010 IEEE International Symposium on Biomedical Imaging: From Nano to Macro.

[59] Pan Y., Christensen G.E., Durumeric O.C., Gerard S.E., Reinhardt J.M., Hugo G.D. Current-and Varifold-Based Registration of Lung Vessel and Airway Trees. Proceedings of the IEEE Conference on Computer Vision and Pattern Recognition Workshops, Las Vegas, NV, USA, 26 June–1 July 2016.

[60] Pan Y., Christensen G.E., Wei Shao S.E.G., Durumeric O.C., Hugo G.D., Reinhardt J.M. Pulmonary Blood Vessel and Lobe Surface Varifold (PVSV) Registration. Proceedings of the 2020 IEEE 17th International Symposium on Biomedical Imaging (ISBI 2020).

[61] Sotiras A., Davatzikos C., Paragios N. (2013). Deformable medical image registration: A survey. IEEE Trans. Med. Imaging.

[62] Bookstein F., Green W. (1993). A Thin-Plate Spline and the Decomposition of Deformations. Math. Methods Med. Imaging.

[63] Kybic J., Unser M. (2003). Fast parametric elastic image registration. IEEE Trans. Image Process..

[64] Rueckert D., Frangi A.F., Schnabel J.A. (2003). Automatic construction of 3-D statistical deformation models of the brain using nonrigid registration. IEEE Trans. Med. Imaging.

[65] Xie Z., Farin G.E. (2004). Image registration using hierarchical B-splines. IEEE Trans. Vis. Comput. Graph..

[66] Shao W. (2019). Improving Functional Avoidance Radiation Therapy by Image Registration. Ph.D. Thesis.

[67] Shao W., Pan Y., Durumeric O.C., Reinhardt J.M., Bayouth J.E., Rusu M., Christensen G.E. (2021). Geodesic Density Regression for Correcting 4DCT Pulmonary Respiratory Motion Artifacts. Med. Image Anal..

[68] Murphy K., Van Ginneken B., Reinhardt J.M., Kabus S., Ding K., Deng X., Cao K., Du K., Christensen G.E., Garcia V. (2011). Evaluation of registration methods on thoracic CT: The EMPIRE10 challenge. IEEE Trans. Med. Imaging.

[69] Kipritidis J., Tahir B.A., Cazoulat G., Hofman M.S., Siva S., Callahan J., Hardcastle N., Yamamoto T., Christensen G.E., Reinhardt J.M. (2019). The VAMPIRE challenge: A multi-institutional validation study of CT ventilation imaging. Med. Phys..

[70] Avants B., Tustison N., Song G. (2008). Advanced normalization tools (ANTS). Insight J..

[71] Ding K., Yin Y., Cao K., Christensen G.E., Lin C.L., Hoffman E.A., Reinhardt J.M. (2009). Evaluation of Lobar Biomechanics During Respiration Using Image Registration. Proceedings of the International Conference on Medical Image Computing and Computer-Assisted Intervention.

[72] Deformetrica Software version 3.0. https://www.deformetrica.org/.

[73] Woodruff P.G., Barr R.G., Bleecker E., Christenson S.A., Couper D., Curtis J.L., Gouskova N.A., Hansel N.N., Hoffman E.A., Kanner R.E. (2016). Clinical Significance of Symptoms in Smokers with Preserved Pulmonary Function. N. Engl. J. Med..

